# A Futuristic Development in 3D Printing Technique Using Nanomaterials with a Step Toward 4D Printing

**DOI:** 10.1021/acsomega.4c04123

**Published:** 2024-08-26

**Authors:** Prachi Agarwal, Vidhi Mathur, Meghana Kasturi, Varadharajan Srinivasan, Raviraja N Seetharam, Kirthanashri S Vasanthan

**Affiliations:** † Manipal Centre for Biotherapeutics Research, Manipal Academy of Higher Education, Karnataka, Manipal 576104, India; ‡ Department of Mechanical Engineering, University of Michigan, Dearborn, Michigan 48128, United States; § Manipal Institute of Technology, Manipal Academy of Higher Education, Karnataka, Manipal 576104, India

## Abstract

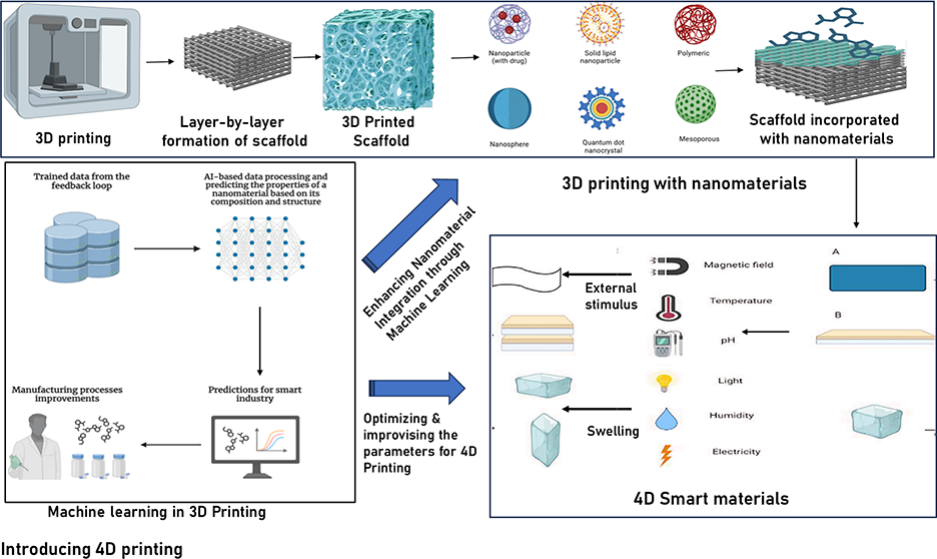

3D bioprinting has shown great promise in tissue engineering and regenerative medicine for creating patient-specific tissue scaffolds and medicinal devices. The quickness, accurate imaging, and design targeting of this emerging technology have excited biomedical engineers and translational medicine researchers. Recently, scaffolds made from 3D bioprinted tissue have become more clinically effective due to nanomaterials and nanotechnology. Because of quantum confinement effects and high surface area/volume ratios, nanomaterials and nanotechnological techniques have unique physical, chemical, and biological features. The use of nanomaterials and 3D bioprinting has led to scaffolds with improved physicochemical and biological properties. Nanotechnology and nanomaterials affect 3D bioprinted tissue engineered scaffolds for regenerative medicine and tissue engineering. Biomaterials and cells that respond to stimuli change the structural shape in 4D bioprinting. With such dynamic designs, tissue architecture can change morphologically. New 4D bioprinting techniques will aid in bioactuation, biorobotics, and biosensing. The potential of 4D bioprinting in biomedical technologies is also discussed in this article.

## 1 Introduction

Biofabrication is an interdisciplinary and potentially multidisciplinary research domain that integrates manufacturing processes to construct models, biomimetics, bioprototypes, and bioproducts at the forefront of bioengineering innovation. It accomplishes this by combining principles, protocols, and practices from engineering, biology, and material sciences.^1^ The domain of biofabrication continues to experience rapid and uninterrupted growth, giving rise to unpredictable scientific developments. Biomaterials are engineered mixtures or pure substances that can function as building blocks, interact with biological systems, recover, regenerate, remodel, redesign, or recreate structural or functional components, and restore, replace, reconstitute, regenerate, or remodel structural or functional components. “Nanomaterials” refers to materials within the dimensional range of nanoscale, typically ranging from 1 to 100 nm in size. Possession of unique properties such as high surface area to volume ratio, quantum effects, and increased reactivity makes application of nanomaterial valuable for various applications in different fields, including tissue engineering and regenerative medicine. These nanomaterials can be engineered to have definite characteristics of improved mechanical strength, electrical conductivity, and biocompatibility, allowing them to interact effectively with biological systems for improving targeted drug delivery, imaging, and tissue regeneration.^2^ Nanobiomaterials interact and get absorbed by cells, thereby triggering cellular reactions. It can activate cellular receptors and guide cells to exhibit specific behaviors, which is why they are also utilized in TE and regenerative medicine. These biomaterials have been synthesized from ceramics, metals, and polymers, encompassing a vast array of substances. They are classified based on their suitability for use in either flexible or hard TE. Multiple nanomaterials can be utilized as carriers for transporting bioactive elements toward the biofabrication processes. Specifically, intelligent nanomaterials, like stimuli-responsive materials designed to sense the environment and react accordingly, will play a significant role in advancing the emerging field of nanobiofabrication. It is anticipated that the advancement of the new nanobioinks will likely transform the process of biofabrication in the near future.^3^

Nanostructured materials have been employed to enhance the properties of biomaterials and have proven to be significant in the biological environment. Advancements in nanotechnology could greatly benefit 3D and 4D bioprinting strategies by meeting the necessary technical specifications for utilizing these advanced materials.^4^ The objective of 4D bioprinting methods is to create and adjust 3D structures by using evolving self-assembly processes that can alter their shapes or functions over time. This is achieved by introducing specific triggers such as chemicals (like pH, salts, and other solution components), biological factors (such as biomolecules and organic compounds), or physical stimuli (including temperature, light, magnetic field, ultrasound, electric field, or osmotic pressure) or through self. Even though 4D bioprinting technology has only recently emerged, leading to the creation of the first smart 3D bioconstructs, researchers in the field are already identifying some initial limitations.^5^ Enhanced smart materials, such as biomaterials and nanomaterials, are necessary for reliable bioconstructs. Additionally, there is a need for precise and efficient methods to establish these innovative biofabrication processes. It is certain that new challenges and obstacles will arise from the research, but the multidisciplinary nature of this field will aid in resolving the issues.

## 2 3D Printing of Nanomaterials

**Figure 1:**
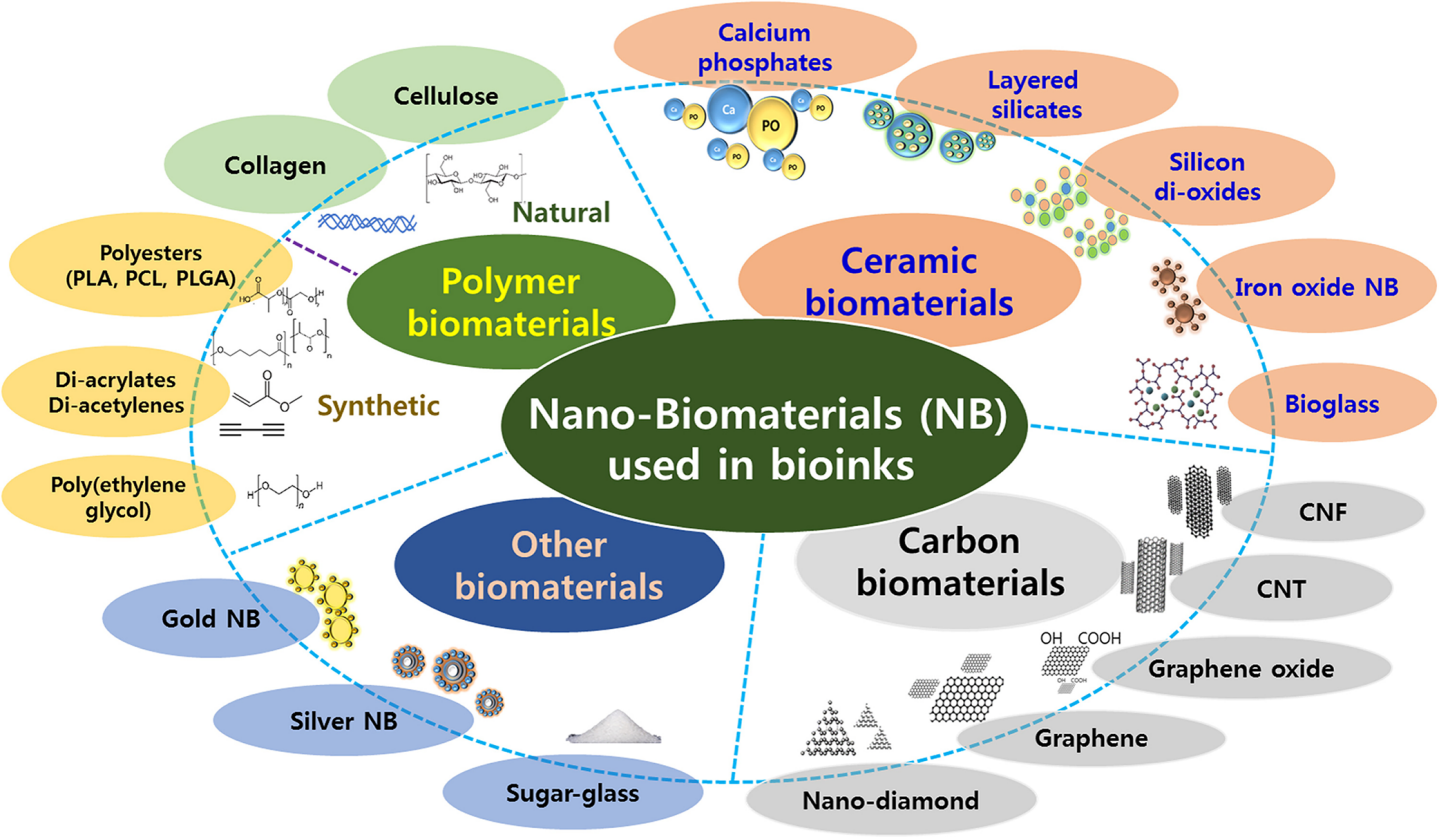
Various nanomaterials incorporated with bioinks for 3D bioprinting NP = nanoparticle, CNT = carbon nanotube, CNF = carbon nanofiber, PLA = poly(lactic acid), PCL = poly(ε-caprolactone), PLGA = poly(lactic-*co*-glycolic acid). Nanobiomaterials for designing functional bioinks toward complex tissue and organ regeneration in 3D bioprinting (reproduced from ref (106) Elsevier).

Customized biomaterials are manufactured via 3D printing technology as a tool to use in TE and RM applications. 3D printing can be performed using a wide range of biomaterials that includes metals, ceramics, polymers, and composites. Nanomaterials combined with 3D printing polymers create novel, adaptable, multifunctional hybrid materials that can be employed in a variety of biomedical applications.^6^ The recent breakthroughs in novel hybrid biomaterials for biomedical applications and 3D printing technology employ nanomaterials including metal, silicon, ceramics, cellulose, carbon, nanocellulose mixes, and others. Some of the poor mechanical properties of current 3D-printed implants may be solved if nanomaterials are employed effectively.^7^ There are 3D-printed scaffolds that have been coated or loaded with nanoparticles for the treatment and averting of disease. This interconnected approach has a number of benefits, including the ability to modify the mechanical properties of 3D printed scaffolds to create a structure that is equivalent to the strength and general makeup of biological tissues and organs, promote cell division and proliferation, and use silver nanoparticles antibacterial properties to prevent the onset of bacterial infection, which can occasionally occur after transplantation.^8^ Additionally, nanomaterials have the unique properties and cutting-edge capabilities needed to alter the biological behavior of existing 3D-printed objects. They have also shown great promise in the treatment of diseases, delivery of medications, and tissue regeneration. Customizing and individualized medicinal products is another advantage of nanotechnology and 3D printing. Increased productivity and the democratization of design are other advantages. Without a doubt, the use of nanomaterials in conjunction with 3D printing technology will offer significant potential for the development of innovative nanocomposites with improved functionality, which will ultimately support a number of medical fields^9^ as depicted in Figure 1.

### 2.1 Silicon Containing NanOmaterials

Nanoclays, nanosilicates, silica nanoparticles (SiNPs), and polyhedral oligomeric silsesquioxanes are silicon-based nanomaterials. These have a strong consequence on the development of nanocomposites that are polymeric in nature. By adjusting the concentration of the silicates, it is possible to tune the characteristics of various polymers by using synthetic and modified silicates. The two different silicon-based nanomaterials in cell-filled 3D bioprinting inks are described below with their applications.^10^

#### 2.1.1 Nanoclays (NC) or Nanosilicates (NS)

Silicon (Si) atoms are tetrahedrally bonded to an octahedrally shared edge of aluminum hydroxide (Al(OH)_3_) or magnesium hydroxide (Mg(OH)_2_) in layered silicates known as nanoclays. Trioctahedral smectite, or laponite nanoclay, is made of layered silicates and inorganic mineral salts.^11^ The positively loaded edge and the negative charge on the surface of the nanoclay dual charge distribution give it the ability to be stable in aquatic environments, thin under shear, encapsulate large amounts of drugs, and improve cell-material interactions. Nanoclays are utilized in various biomedical applications and medicinal delivery due to their distinctive features like form, high surface area to volume ratio, charge, and compatibility to biological systems.

##### 2.1.1.1 Experimental Studies

Biomaterials such as gelatin and alginate encapsulated with nanosilicates are used for bioprinting the bone constructs using rat bone marrow mesenchymal stem cells (rBMSCs). While printing, NS decreases the resistance flow at a high shearing speed and prevents any harm being done to the cells that are encapsulated by shear stress.^12^ rBMSCs are promoted to differentiate osteogenically when the NS are embedded into the 3D printed scaffolds. These cells demonstrated an improvement in the expression of genes and proteins, developed mineralization, and enhanced bone production made possible by biologically active magnesium (Mg^2+^), orthosilicic acid, and lithium (Li+). The deterioration of printed scaffolds may be the cause of the 3D nanocomposite scaffold’s effective bone mending capacity *in vivo*.^13^

Similar to skeletal regeneration, bioinks should promote vascular network infiltration, accelerate cell proliferation, drive cell differentiation, and keep encapsulated cells visible. EBB was used to create a 3D structure which has the biomimetic properties of the bone which uses GelMA as the bioink with cells that are osteogenic and vasculogenic in nature.^14^ Human umbilical vein endothelial cells (HUVECs) and human mesenchymal stem cells (hMSCs) were used to design a perfusable blood artery inside of a quickly degradable GelMA hydrogel. GelMA hydrogel was designed as such that it encourages the differentiation of hMSCs with osteoblastic lineage. To do that hydrogel was laden with hMSCs along with silicate nanoplatelets. For encouraging vascular propagation, GelMA was attached chemically with different gradient concentrations of vascular endothelial growth factor (VEGF).^15^

The perfused construct secreted much more bone like ECM than the nonperfused one, demonstrating favorable impact on the development of osteoblasts and formation of the bone. Quantifying and immunostaining gene expression of osteogenic markers osteorelated proteins osteocalcin (OCN)/cluster of differentiation (CD31) and runt-related transcription factor-2 (RUNX2) /CD31, supported the proposition that silicate nanoplatelets caused differentiation of hMSCs into osteoblastic lineage.^16^

#### 2.1.2 Silica Nanoparticles (SiNPs)

These are widely used in bioimaging, catalytic, chemical processes, and the transport of drugs and genes because of their unique hydrophilic qualities, including their regular spherical form, huge area of section, and strong heating and automated capabilities. SiNPs may be produced as homogeneous particles ranging in size from 50 to 2000 nm, which is essential for the nanocomposite’s mechanical strength.^17^ The biological uses of SiNPs can be expanded by surface functionalizing and bioconjugating them with reactive functional groups as-synthesized. In order to create dynamic covalent connections for high-precision bioprinting without compromising biocompatibility, we altered both SiNPs and alginate. The combination of oxidized alginate-containing polymeric ink (OxALG) and aminopropyl-modified SiNPs (NH2-SiNPs) demonstrated improved shear-thinning capabilities and good structural fidelity. The compressive and tensile moduli of the nanocomposite ink were greater. Even though the bioprinted gels helped chondrocytes mature both *in vitro* and *in vivo*, neither host cells nor blood vessels invaded the gels after they were cultivated.^18^

### 2.2 Ceramic-Based Nanomaterials

Due to their exceptional biocompatibility and osteoconductivity, ceramics are strong candidates for complicated repairing of the tissues and their regeneration, particularly for fixing the voids and flaws of the bone.^19^ The 3D bioprinting process has been used to produce a variety of ceramic-based nanomaterials, such as calcium phosphates (CaP) and bioactive glasses. Tissue regeneration of bone and cartilage can be done with the help of using ceramic nanoparticles that are introduced into the hydrogels containing cells. These slowly degrading hydrogels provide a sustained architectural support and osteogenic characteristics that resemble the natural minerals of the bone.^20^

The ionic-exchange kinetics and protein absorption of nanoparticles are determined by their composition, surface chemistry, and topography, which, in turn, influences cellular activities. On the nanoapatite crystal surfaces, the cells adhered and showed a healthy spreading polygonal shape. The nanoapatite structure encouraged the growth of stem cells.^21^ By increasing the expression of osteogenic genes including BMP2 and RUNX2, which result in the production of ECM components and biomineralization, the inclusion of nano-CaP enhanced osteogenic cell development.^22^

Nanostructure, nanocrystallinity, and nanoscale roughness are features of biodegradable and bioactive glass nanoparticles (BGNP) and nano-CaP properties that have a significant impact on the interaction between cells and materials. With the capacity to absorb and sustain the leasing of osteogenic GFs to induce osteogenesis, they offer additional binding sites for cell adhesion. Interactions amid biomolecules, different cells, BGNPs, and nano-CaP help in induction of the osteogenic pathway and being anchored to the cell membrane receptors.^23^

#### 2.2.1 Bioactive Glass Nanoparticles

The characteristics that make these BGNPs a strong candidate to be used for bone tissue engineering is that, first, they have customized configurations, have controlled resorbability when confronted to any physiological circumstances, and ability to illicit suitable cellular responses in bones and other tissues when it comes in contact with physiologically fitting ions.^24^ Hydrogels like alginate dialdehyde gelatin mixing with BGNPs and BGNPs that are intoxicated with strontium (BGNPsSr) under the circumstance of simulated bodily fluid induce formation of apatite layer that is bonelike on the surface that promotes cell adhesion and proliferation. Sr^2+^ ions are acknowledged for inducing osteogenesis and growth of the bones, which is done *in vivo*, and were controlled by BGNPsSr. With a larger concentration of BGNPs, the gels became more viscous. The inclusion of BGNPs lowered gelation time from 60 to 15 min.^25^

#### 2.2.2 Calcium Phosphate Nanoparticles (CaP)

These groups of compounds include calcium ions (Ca^2+^) and inorganic phosphate anions. CaP has been widely employed for tissue regeneration applications as it provides good osteoconductivity. HAp is an effective bone regeneration replacement material. To create a tunable hydrogel composite, different amounts of HAp were added to alginate and gelatin precursors. The new composite bioink combines the superior cell viability of alginate, the increased structural stability of gelatin, and the outstanding osteoinductivity of HAp, making it an appropriate candidate for bone constructions via 3D bioprinting. In another experiment, 5% HAp particles were mixed with methacrylated gelatin (MeGel) and methacrylated hyaluronic acid (HAM) with human adipose-derived stem cells (hASCs) being encapsulated for bone tissue engineering application.^26^ Viscosity and the printing ability of the hydrogel are increased when HAp is added. During culture, scaffolds that were printed show stabilized characteristics for over 28 days, which demonstrates that the structure has good integrity. Production of bone matrix was observed to be tough when the cells are encapsulated, and there is an increase in the osteogenic markers alkaline phosphatase (ALP) and osteopontin (OPN).^27^ Protein absorption, cell adhesion, and integrin binding were all enhanced by the HAp-modified hydrogel, followed by the activation of intracellular pathways and osteogenic differentiation. To improve the mechanical and structural characteristics of gelatin and alginate hydrogels, HAp was added. The composite hydrogel outperformed pure gelatin and alginate in criteria such as retention of the shape and printing ability. The inclusion of HAp reduced gelatin and alginate’s water swellability making it tougher when an external strain is applied. Rate of scaffold degradation slowed as Hap increased, giving ample room for cell expansion and proliferation.^28^

### 2.3 Cellulose-Based Nanomaterials

Nanocellulose is obtained from natural cellulose and has a 1D nanoscale size. There are three varieties of nanocellulose: nanofibrillated cellulose (NFC), nanocrystalline cellulose (NCC), and bacterial nanocellulose (BNC). Characteristics that distinguish nanocellulose from other nanomaterials are chemical reactivity of the surface, crystallinity, distinctive shape, dimensions, high specific surface area, automated reinforcement, and biocompatibility.^29^ As the nanocellulose imitates the fibril network of the ECM, it is a significant reinforcing material for 3D bioprinting as it provides good mechanical stability, suitable environment for the cells, and shear thinning abilities with good quality of printing. Nanocellulose being encapsulated with cells in the hydrogel has a variety of 3D bioprinting applications, including cartilage, tendon, bone, skin, face, liver, and others.^30^

#### 2.3.1 Nanofibrillated Cellulose

Long, intertwined fibrils consisting of cellulosic particles that are both crystalline and amorphous in nature are NCF which can also be termed as cellulose microfibrils (CMF). They are approximately 1 μm in length and 5–60 nm in diameter. A high ratio between the maximum horizontal and vertical length of NCF is responsible for the gel-like consistency it has in aqueous mediums, whereas the surface-modified groups facilitate the opportunities for its functionalization.^31^ There was an experiment where the bioink is encapsulated with adipocytes with the use of nancellulose and hyaluronic acid.^32^ The bioinks that are incorporated with cell suspensions were extruded from the cartridge. When the circumstances of the cell culture is well-maintained, the cellulosic nanofibrils make porous networks. There is an upregulated expression of adipogenic marker genes like peroxisome proliferator-activated receptors (PPAR) and fatty acid-binding protein 4 (FABP4) in such 3D printed culture systems along with a stronger expansion of MSCs into adipocytes. The mixture of nanocellulose and hyaluronic acid is commercially accessible, and their compatibility with the process of cell encapsulation is an advantage to form a bioink.^33^

#### 2.3.2 Nanocrystalline Cellulose

These are extracted from the crystalline regions of the cellulosic fibers. The structures have a diameter of 3–10 nm, and the length of these structures when kept in an aqueous medium is around 50–500 nm. NCC was preferred to strengthen complex matrices with mechanically anisotropic topographies because it exhibited relatively high rigidity and ordered alignment in the liquid crystal phase. NCC has been researched for a variety of applications and has several advantageous features.^34^

Conjugating 2,2,6,6-tetramethylpiperidinyloxy (TEMPO)-modified nanocrystalline cellulose (mNCC) to the methacrylated gel (MeGel) backbone generated mNCC-MeGe (mNG) composite hydrogel. Encapsulated human-adipose-derived MSC (HADMSC) in mNG hydrogels was bioactive and disseminated more widely. They took on a dormant fibroblastic phenotype and demonstrated phenotypic features present in the heart valve’s spongiosa.^35^ Because of their improved mechanical characteristics and environment for GAG deposition as well as their decreased susceptibility for calcification, the hydrogels demonstrated nonlinear biomechanics and might be beneficial for designing the fibrosa layers. Furthermore, after 7 days of culture, the 3D bioprinted construct of the mNG biomaterial with HADMSC demonstrated viable cells. This research established the capability of employing mNG as a biomaterial to possibly build many layers of the heart valve.^36^

#### 2.3.3 Bacterial Nanocellulose Fibrils

Culture media like glucose and xylose can be used for the formation of bacterial nanocellulosic fibers, which have structural similarities to that of bacteria. Ribbons with an interconnected structure and dimensions of 100 nm in diameter and 100 m in length are produced by the fibrils. BNC has excellent water retention, high crystallinity of CMF, high levels of polymerization up to 10,000, good mechanical properties, and flexibility.^37^ In an experiment, the aqueous counter collision (ACC) technique was used because, when applied to BNC, it successfully dissociates weaker intermolecular interactions without any chemical modification, leading to degradation and fibril disentanglement. Sixty days following the implantation of human cells in naked mice, excellent chondrocyte proliferation capability and tissue integration were observed. The study suggested that the BNC produced by the novel ACC disentanglement technique is extremely recommended for the 3D bioprinting applied in reconstructive surgery.^38^

### 2.4 Metal-Based Nanomaterials

Electrical conductivity, durability, and magnetic behavior are characteristics of metal-based nanoparticles. These are included into a range of a variety of biomaterials to improve functionality and printability for tissue engineering applications. In the presence of a magnetic field, iron and iron oxide nanoparticles (IONPs) exhibit magnetic activity which were added to hydrogels or injected into cells in the form of nanomaterials to provide MRI or computed tomography (CT) images to simplify procedures and the tracking of bioactive components inside a 3D tissue construct.^39^ Due to their ease of manufacture and modification, range of aspect ratios, and biocompatibility, gold nanomaterials (GNMs) such as gold nanowires (GNWs), gold nanoparticles (GNPs), and gold nanorods (GNRs) offer promise in biomedical applications. They were mixed with bioinks acting as structural cues to align the cells or to simulate the electrical characteristics of muscle tissues. Strontium–carbonate nanoparticles were reported to improve the osteogenic differentiation by incorporating into GelMA hydrogel.^40^

#### 2.4.1 Iron and Iron Oxide Nanoparticles

Cells that have undergone phenotypic development must mimic their ECM in certain microenvironments. For instance, the three zones that made up the articular cartilage had different chondrocyte morphologies and ECM compositions. The superficial zone joint is represented by collagen fibers that are horizontal. Collagen fibers were dispersed in the intermediate zone and vertically oriented in the deep zone to sustain the mechanical load of the joint. An enhanced bioprinting method was demonstrated by adding a magnetic-based fiber alignment mechanism into a drop-on-demand (DoD) 3D bioprinter in order to produce multilayered tissues that closely mimic real tissues.^41^ Agarose and type I collagen hydrogel mixes (Col I) were combined with streptavidin-coated iron nanoparticles (INPs). The implanted INPs moved unidirectionally during printing in the presence of a magnetic field, aligning the collagen fibers in parallel. By up to 20% of the compressive strain, the unidirectional fiber alignment increased the hydrogel’s compression modulus. Multilayered bioink structures that were printed with different fiber orientations outperformed single-layered structures in terms of chondrogenesis.^42^

#### 2.4.2 Gold Nanomaterials

Myoblast satellite cells fuse and differentiate into the long fibrous bundles of multinucleated myotubes that make up the ECM of skeletal muscle. In order to promote cell alignment and proliferation, gold nanoparticles (GNs) were added to collagen-based bioink, simulating the anisotropic electrical microenvironment of genuine muscle tissues.^43^ While printing, shear stress induced due to the flow of the ink through a microsize nozzle was used to align the GNWs in the collagen bioink in the extrusion direction. The GNWs-loaded scaffolds demonstrated reduced fibrosis and fewer infiltrated inflammatory cells than those without GNWs structures after being implanted in the temporalis muscle flap, which is ideal for repairing muscle tissues.^44^

#### 2.4.3 Strontium–Carbonate Nanoparticles

The treatment of BTE frequently used strontium. The chemically identical strontium ions to calcium ions have been shown to promote bone growth, inhibit bone resorption, and integrate with bone HAp. An appropriate concentration of strontium–carbonate (Sr) nanoparticles was added to 5% GelMA that was packed with hMSCs. The created bioink Sr-GelMA displayed a noticeably higher viscosity when compared with pure GelMA, which enhanced printability and structural integrity. The printed scaffolds held up well in culture for a period of 28 days.^45^ Strontium (Sr) nanoparticles were added to the cell-filled bioink to maintain the high cell viability (>95%) of the encapsulated hMSCs. Increased expression of ALP, OCN, and COL I, as well as the emergence of mineralized nodules with uniform distribution inside the bioprinted structures, are further indications that it promoted osteogenic differentiation of hMSCs. The successful printing of a 3D Sr-GelMA construct with high shape retention and osteogenic potential illustrates the promising potential of strontium-based nanocomposite bioinks for bone tissue regeneration.^46^

### 2.5 Carbon-Based Nanomaterials

Small surface areas, unique optical features, strong thermal conductivity, and high mechanical strength of carbon-based nanomaterials have made them popular in biomedical applications such as drug delivery, reinforcing additives, and cellular sensors. Carbon-based nanomaterials are widely employed in several industries, including biomedicine. Below is a discussion of how carbon nanotubes (CNTs), graphene (G), and graphene oxide (GO) are used in cell-filled bioinks for 3D bioprinting.^47^

#### 2.5.1 Carbon Nanotubes

CNTs are divided into single-walled (SWCNTs) and multiwalled (MWCNTs) types depending on the number of layers. MWCNTs are available in lengths up to several micrometers and diameters ranging from 2 to 100 nm. They have been employed in 3D bioprinting of flexible electronics, specifically patterning encapsulated cells, vasculature, and cardiac tissue constructions due to their unique structure and characteristics.^48^ CNTs have much better tensile strength and elastic moduli than the other materials utilized. CNT size, shape, surface roughness, and surface area physically approximate collagen fibers, in contrast to polymeric fibers like PCL, which lack a 3D network to support and direct cell proliferation, differentiation, and communication.^49^

#### 2.5.2 Graphene and Graphene Oxide (GO)

The large surface area, high mechanical flexibility, and potential for chemical functionalization of graphene (G) have generated significant interest in the biomedical sector. GO is created by the oxidative exfoliation of graphite and is an atomically thick carbon sheet. It has a variety of chemical groups, such as hydroxyl, carboxylic, and epoxy groups, which enables it to interact with a variety of molecules. From nano- to a few microns, the size ranges. For the tissue engineering of cartilage, bone, and nerves, graphene and GO have been used as bioink additives.^50^

In a study, it was shown that 3D bioprinting technology was used to make cell-filled GO/alginate/gelatin composite scaffolds that mimicked bone. By increasing the expression of ALPL, BGLAP, and PHEX, the scaffolds promoted osteogenic differentiation while also maintaining excellent fidelity, cell protection, and cell viability. Osteoblastic/osteocytic cell differentiation and ECM mineralization are strongly influenced by the concentration of GO.^51^

### 2.6 Other Nanomaterial Type

There are numerous additional types of nanomaterials, including upconversion, lipid-based, polymeric, and composite nanomaterials. Specialized digitally guided controlled microstructures are required for the fabrication of polymer nanocomposites; this area must be explored further for tissue engineering applications. By applying the proper pre- and postprocessing methods and functionalizing the micro surfaces of 3D printed scaffolds, the potential toxicity or negative impacts of nanocomposites must be reduced. The primary goal of fusing ideal build characteristics with tissues for effective healing may be tackled once the basic concerns with processes and products have been addressed.^52^ Interdisciplinary teams of scientists, engineers, and medical experts now have a multitude of tools at their disposal for advancing medical technology as a result of the confluence of nanotechnology, polymer engineering, and additive manufacturing. In a rat model utilizing sodium alginate, bioglass, and modified chondroitin sulfate, a triple cross-linked hydrogel with acyl-hydrazone, noncovalent, and Diels–Alder click covalent cross-linking generated effective cranial bone repair, the creation of 3D printed, highly conductive objects with silver nanowire and carboxymethyl cellulose. Gold nanorod was dissolved in GelMA bioink to create a bioprinted heart architecture using neonatal rat cardiac cells.^53^ High electrical conductivity of the nanorods promotes the coordinated contraction of the construct and enhances cell–cell communication. In the primary hepatocyte cell-laden hydrogels, sugar-glass is employed to 3D print large complicated structures and build a sacrificial linked 3D vascular lattice. It is possible that this is a nanoparticle that uses nanotechnology to transport biomolecules in a regulated way. Over the past several years, there has been a considerable rise in the quantity of research on *in vitro* and *in vivo* tissue engineering studies to manage the interactions between 3D printed scaffolds and the intracellular matrix. All of them indicate an area that is expanding quickly and has the potential to significantly influence medicine and healthcare in the ensuing decades, including personalized intervention. As nanocomposites and additive manufacturing revolutionize tissue engineering and regenerative medicine, more novel printable biopolymers and biocompatible nanomaterials are anticipated to be developed. This is because the need for implants, transplants, and biorepairs has grown along with life expectancy.^54^ There is a list of several nanomaterials, their functional characteristics, and uses in Table 1.^55−62^

**Table 1:** Overview of Nanomaterials Combined with 3D Bioprinting and Their Application in the Biomedical Field

Type of nanomaterial	Functional Property	Biomedical application	References
Silicon-based			
(1) Nanoclays	-Nontoxic	-Osteogenic and angiogenic differentiation	(55)
	-No immunogenic response	-Drug delivery	
	-Improves cell adhesion property of hydrogel	-Biosensors	
(2) Silica nanoparticles	-High specific surface area	-Bone tissue engineering	(56)
	-Have low toxicity	-Resist postsurgical pathogenic infections	
	-Good thermal instability	-Used for maintaining the sustained release of drugs	
Ceramic-based			
(1) Bioactive glass	-These nanomaterials are hard and brittle and good biocompatibility, hydrophilicity, osteoconductivity	-Bone tissue engineering	(57)
		-Vascularization in bone grafts	
		-Dental pulp tissue regeneration	
(2) Calcium phosphate	-Antibacterial properties	+HAp^a^: Skeletal tissue engineering; +Nanocrystalline HAp; +PLGA^b^ + TGF-β1^c^: osteochondral differentiation ligament and cartilage	(58)
Carbon-based			
(1) Carbon nanofiber	-Remarkable mechanical properties	Can be used for the preparation of Meniscus scaffold	
	-Imparts electrical conductivity		
(2) Carbon nanotube	-Improves cell–cell interaction in the 3D printed structure; antioxidant activity	-Cancer treatment	(59)
		-Bone and cardiac tissue engineering	
		-Articular cartilage tissue engineering	
(3) Bacterial nanocellulose fibrils	-Excellent biocompatibility	-Dental and bone tissue engineering, connective tissue repair	(60)
(4) Graphene oxide	-Good water holding capacity	-PLGA-based ink: neural tissue engineering	
(5) Graphene	-Antimicrobial and antibiotic effect; neural stem cell adhesion; proliferation and differentiation	-Dental implants	
		-For rectification of mandibular defects	
Metal-based			
(1) Metal nanoparticles/nanotubes	-Tissue reconstruction	-Cardiac tissue engineering	(61)
-Gold nanobiomaterial	-Enhance cardiac cell adhesion	-Plasmonic gold nanoparticle releases two miRNAs from the expression of angiogenic factors	
-Silver nanobiomaterial	-Improved antimicobial effect	alginate hydrogel + calve chondrocyte: Bionic ear	
	-Re-epithelialization	-Diabetic wound healing	
	-Wound closure ability		
(2) Metal oxide nanoparticles/nanotubes	-Antibacterial nanoparticles	-Superparamagnetic iron oxide is used for drug delivery	
	-High mechanical strength	-TiO_2_^d^ is used for dental implants and bone regeneration	
	-Promotes osteointegration	-3D printed TiO_2_ nanotubes implants contains anticancer drugs and ligands that induces apoptosis	
	-Chondrocyte cell proliferation	-ZnO^e^ and MgO^f^ for wound healing	
(3) Liquid Metal	-Low melting points	-Ga^g^ used in drug delivery, anti-infection, tumor therapy	
	-Large surface tension	-Galinstan which is an alloy of indium, tin, and Ga used for printing medical devices for the purpose of electrical stimulations of the nerve	
	-Good thermal conductivity		
	-Supercooling effect		
	-High electrical conductance		
Polymer-based			
-Natural biomaterials	-Good moisture absorber	Delivery of biomolecules or pharmacologically active compounds to the cell in a controlled manner	(62)
(1) Cellulose	-High permeability of oxygen		
(2) Collagen	-Biodegradable		
(3) Alginate	-Biocompatible		
-Synthetic biomaterials	-Can adsorb metal ions	Can encapsulate different molecules that includes drugs, antigens of the vaccines, growth factors, proteins, etc.	
(1) Polyesters	-Improve pharmacokinetic property of drug		
(2) Diacrylates			
(3) Diacetylenes			
(4) Polyethylene glycol			

a HAp: hdroxapatite.

b PLGA: poly(lactic-co-glycolic acid).

c TGF-β1: transforming growth factor-β1.

d TiO2: titanium(II) oxide

e ZnO: zinc oxide,

f MgO: magnesium oxide.

g Ga: gallium.

## 3 Influence of Machine Learning on 3D Printing with Nanomaterials

Machine learning is changing additive manufacturing processes and the associated materials. Machine learning algorithms can assimilate enormous data from the whole additive manufacturing process, analyze it, and hence develop the capability for real-time monitoring and control.^63^ This capability enables the optimization of those printing parameters that eventually lead to improvements in product quality and reductions in production costs. ML is based on different algorithms, and there are four types of ML algorithms, namely, supervised learning where the system learns from a set and labeled data set then proceed to make predictions when a new data set is come across,^64^ unsupervised learning helps in understanding the data set without a labeled output and making predictions for complex data without an involvement of humans,^65^ semisupervised combines both supervised and unsupervised learning and training is done on both the types of data,^66^ and reinforcement learning is based on the trial and error process keeping the parameters and end values on point to achieve the result.^67^

**Figure 2:**
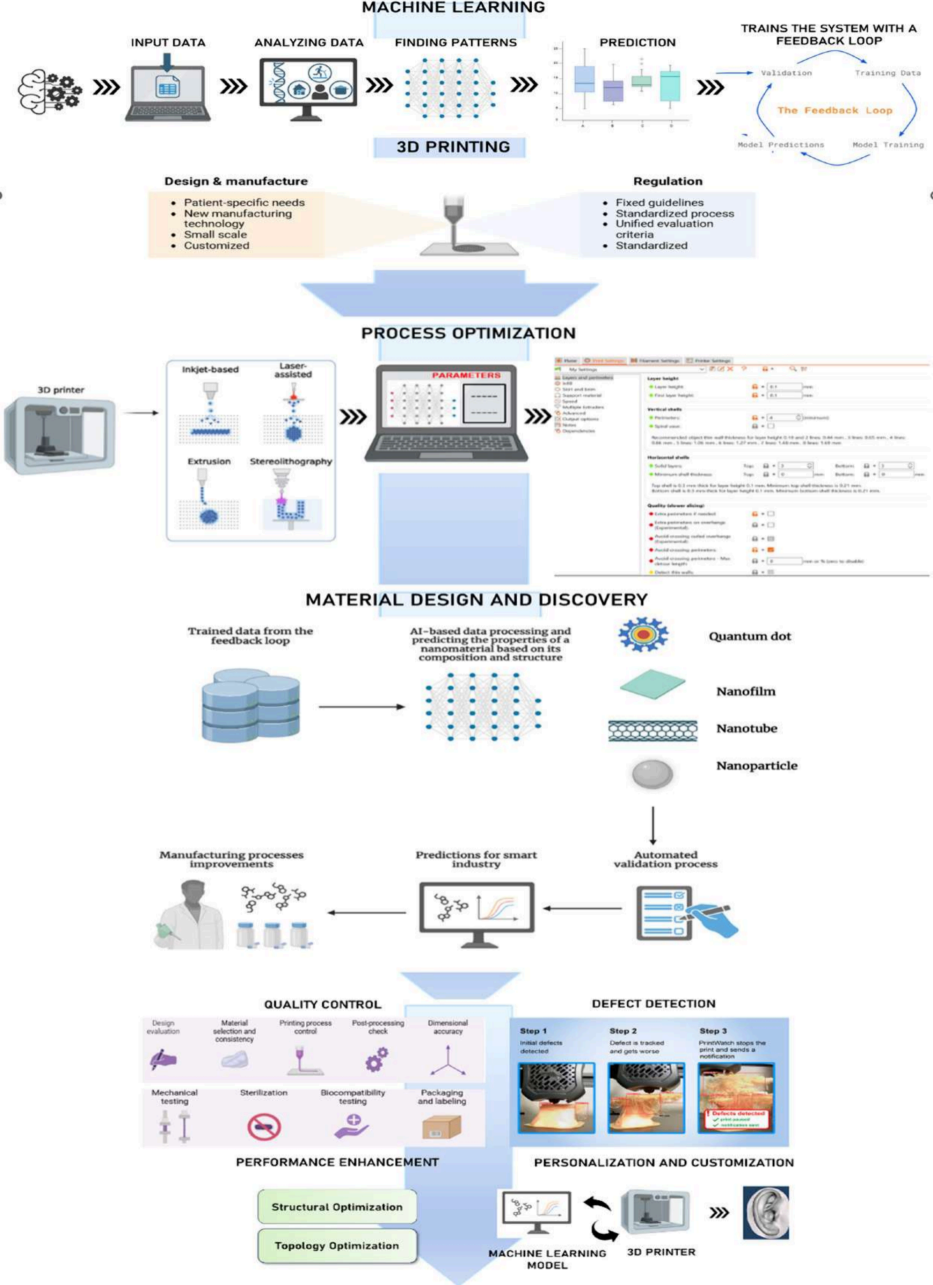
Smart additive manufacturing: A machine learning-driven approach.

When integrated with additive manufacturing (AM), ML offers new opportunities to enhance the entire manufacturing process. This includes advancements in material formulation, design optimization, process refinement, and quality control. The combination of ML and AM has the potential to transform the design and production of AM-printed parts. By leveraging the extensive data generated during AM, ML algorithms can gain deeper insights into the processes, leading to optimized designs, accurate predictions of material properties, and improved production quality. For 3D printing with nanomaterials, ML is utilized to study the compositions of nanomaterials such as strength, conductivity, and flexibility. The structures and the geometries are optimized by using the properties of the nanomaterials that helps in reducing the extensive experimental testing and minimizing the material usage. The temperature, printing speed, and layer thickness can be predicted and adjusted via ML to integrate the optimized nanomaterials also illustrated in Figure 2.

Such ML technologies as represented by neural networks and machine learning make it possible to predict probable defects and dynamically adapt the printing process for highly increased precision and reliability of 3D-printed parts.

Another critical area is material development, where machine learning can successfully be utilized in discovering and designing new materials with the desired properties. Analyzing data from prior experiments and simulations, ML models may see patterns and correlations that elude human researchers. It rapidly tracks the development of advanced materials with specific mechanical, thermal, or electrical properties sought for various applications. Besides, it can help optimize the composition and processing conditions of the materials effectively to attain the desired performance characteristics.

**Figure 3:**
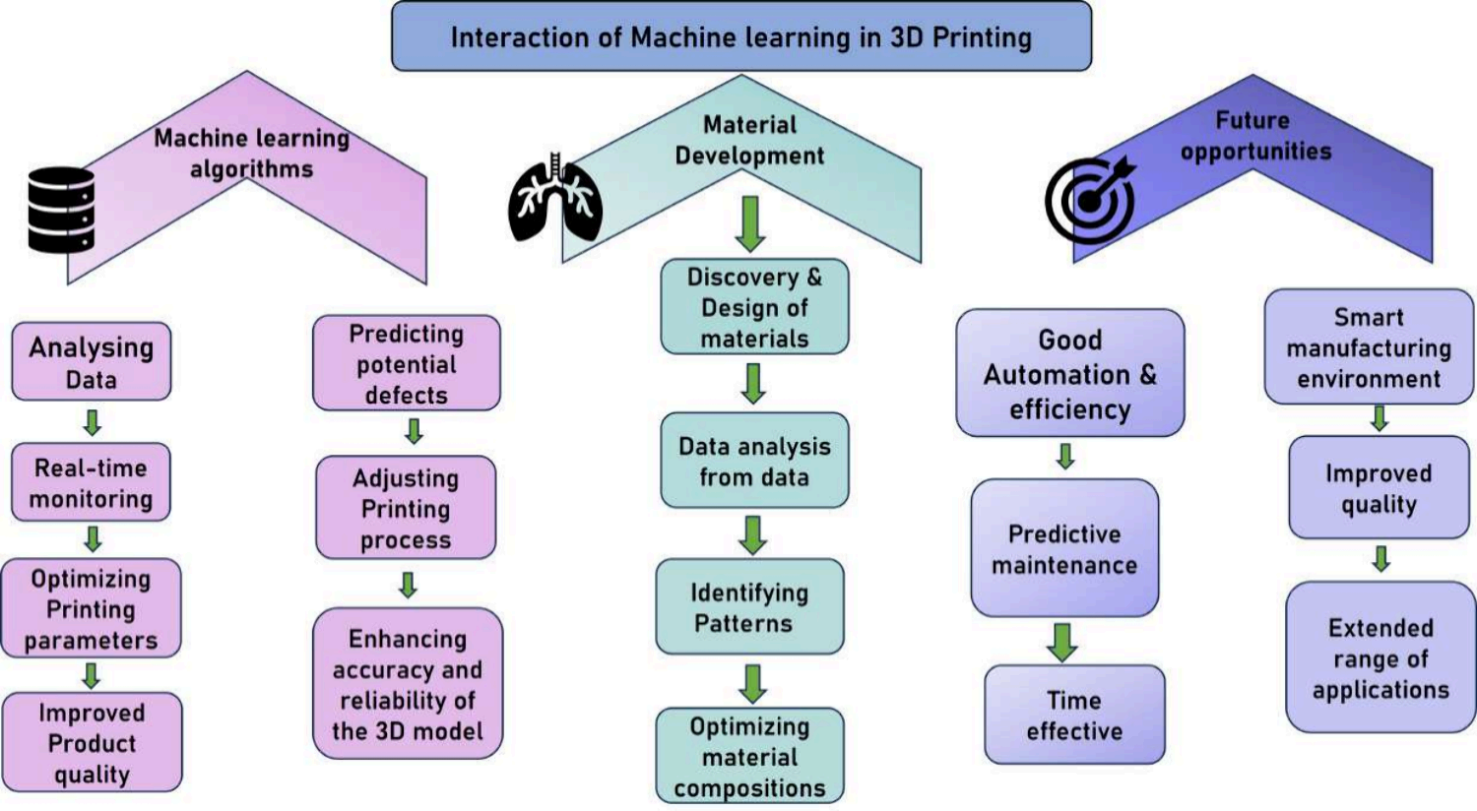
Integration of machine learning and 3D printing workflow.

The future opportunities for machine learning in additive manufacturing have a wide spectrum. As these algorithms continue to evolve, a close integration with the AM technologies is observed, which can result in greater levels of automation and efficiency. Predictive maintenance powered by ML can minimize machine breakdown by predicting equipment failures. Further, in combination with other emerging technologies such as IoT that can lead to an efficient manufacturing environment, ML will support human decision-making, continued learning, and economy in productivity. These developments will improve the quality and performance of 3D-printed products while increasing the range of additive manufacturing applications in industries like aerospace, healthcare, biomedical, and automation. The current and plausible futuristic approaches are illustrated in Figure 3.

## 4 4D Printing

**Figure 4:**
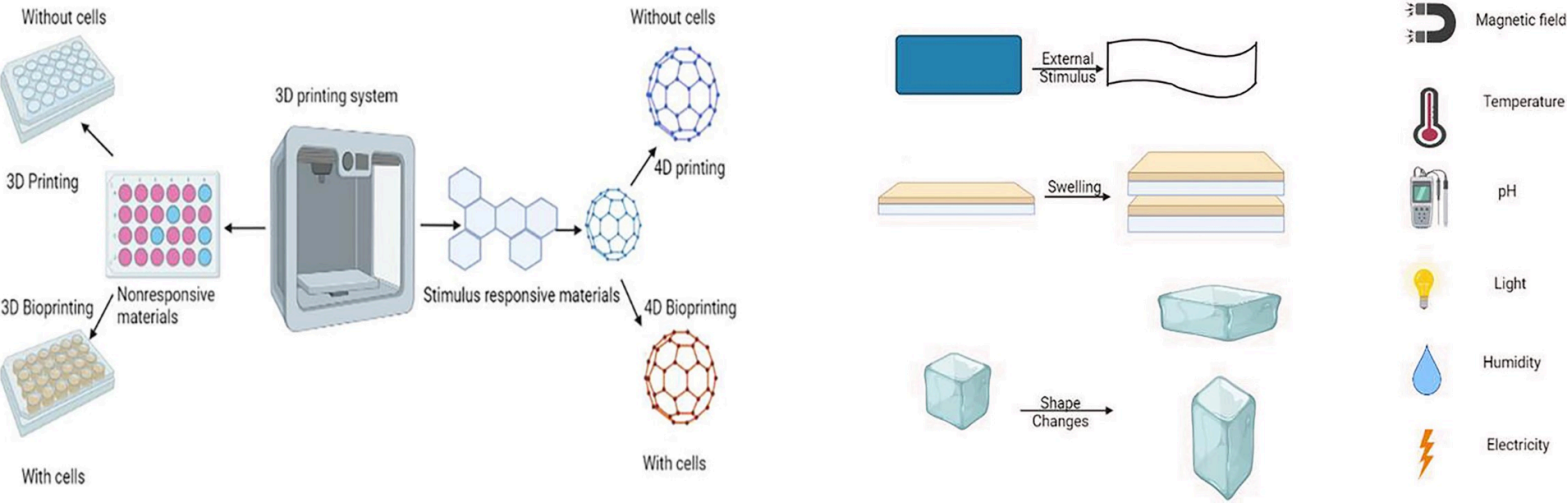
(A) Schematic representation of 3D printing and 4D printing. (B) Types of stimuli for smart materials and response. Reproduced from (68) under Creative Commons CC BY license.

4D printing is an extension of 3D printing with an inclusion of time as the fourth dimension and utilization of smart biomaterials that change size or shape of the structure in response to external stimulus like light, heat, pH, etc.^68^ This technique was first demonstrated by Tibbits et al., at a TED conference where the printed object transformed over time.^69^ The fundamental characteristic of this technology is that it is not static and that it can be preprogrammed to change shape with time. 4D printing is capable of self-assembly and self-repair of the printed object.^70^ 4D printing requires a 3D printer, stimulus, stimulus-responsive material, and mathematical modeling for predictable and targeted evolution of structures over a period of time. 4D printing offers some advantages such as reduction in cost and number of components used in an assembly line. Figure 4 shows the schematic representation of 3D and 4D printing.

### 4.1 Laws of 4D Printing

The laws for 4D printing were formulated to understand the shape changing behavior of the 4D printed structures. The first law states that “all the shape changing behaviors such as coiling, curling, twisting, bending, etc. of multimaterial 4D structures are due to the relative expansion between active and passive materials”. The second law states that, “there are four physical factors behind the shape changing ability of all multi-material 4D structures, i.e., mass diffusion, thermal expansion, molecular transformation, and organic growth”. The third law states that, “time-dependent shape-morphing behaviour of nearly all multi-material 4D printed structures is governed by two “types” of time constants”.^71,72^

### 4.2 Materials in 4D Printing

4D printing utilizes smart materials that can change shape and/color, and these smart materials include shape memory polymers, magnetic shape memory alloys, electro-responsive polymers, shape memory alloys, smart inorganic polymers, temperature responsive polymers, photoresponsive polymer, and electroactive polymers.^73^ Hydrogels are materials that respond to water (moisture). Hydrogels are capable of swelling or expanding their size to up to 200% of their original volume in the presence of water. Hydrogels are highly compatible for bioprinting and have great stretchable properties and good ionic conductivity. However, they have a slow reversal response. A major challenge in smart materials that are used for 4D printing is for the structure to get back to its original form in a controlled manner. The hydrophobic photoinitiators used have low solubility, and the hydrophilic ones are not efficient with hydrogels. Yangyang et al. synthesized a type of microemulsion that can be added to the commonly used hydrophobic photoinitiators which led to high strength and stretchability of the hydrogel along with excellent water-activated shape memory properties.^74^ Lai et al. demonstrated a versatile strategy to develop a hydrogel that is used for 4D printing to create a heterogeneous structure without using chemical reactions. This was done by blending alginate and methylcellulose. This composite showed great rheological properties, printability, and shape fidelity of printed structures.^75^ Guo et al. developed a hydrogel for 4D printing which was fabricated by polymerizing acrylamide in the agarose matrix containing laponite. Their study showed that laponite improved the shear-thinning behavior of the ink, had excellent stability after printing with exceptional mechanical properties of ink as compared to both agarose and polyacrylamide hydrogels.^76^ Hydrogels that respond to heat, light, pH, magnetic field, acoustic field, electric field, and biological factors also exist,^77^ and a few examples are summarized in Table 2.^80−94^ Some polymers that undergo shape morphing effect when the temperature is altered are poly(ethylene glycol) (PEG), poly(N-isopropylacrylamide) (PNIPAM), poly(N-vinylcaprolactam) (PNVCL), gelatin and collagen along with some shape memory polymers like poly(caprolactone triol) (PCL-T), poly(ε-caprolactone) dimethacrylate, acrylated epoxidized soybean oil (AESO), polyurethane (PU), and poly (lactic acid) (PLA).^78^ Liu et al. developed a sprayable thermosensitive hydrogel used for treatment of skin injury in which a sprayable adhesive containing Pluronic F127, zinc, and metformin (ZnMet-PF127) was used. The study found that this adhesive promoted skin healing as it showed improvement in cell proliferation, angiogenesis, and collagen formation.^79^

**Table 2:** Smart Materials in 4D Printing

Stimuli	Fabrication method	Material	Cells	Application	Ref
Heat	Extrusion	Nanothylakoid, poly(N-isopropylacrylamide)	–	Developed artificial breathing actuator	(80)
	Extrusion	Poly(N-isopropylacrylamide), polyacrylamide	–	Implants, soft robotics	(81)
	Extrusion	Polyurethane	hMSCs (human mesenchymal stromal cells)	Time controlled stimulus on cultured cells	(82)
Light	Stereolithography	PEGDA^a^	C2C12 murine myoblasts	Optogenetic muscle ring-powered biobots	(83)
	Extrusion	Alginate/GelMA, alginate/polydopamine	HEK293 cells	Skin, cartilage, and cardiac tissue	(84)
	Extrusion	Poly(ethylene glycol)	–	Direct encapsulation of cells within printable microgels	(85)
pH	Extrusion	Gelatin/chitosan	Hepatocytes, HUVECs^b^	Fabrication of vascular networks in liver scaffolds	(86)
	Direct ink write	Poly(alkyl glycidyl ether) with methacrylate groups	–	Multistimuli responsive hydrogels	(87)
	Hot melt extrusion	poly(2-vinylpyridine)	–	Multistimuli responsive hydrogels	(88)
Magnetic field	Fuse deposition	Fe_3_O_4_^c^/PCL^d^	Human bone marrow stem cells	Bone tissue engineering	(89)
	Fuse deposition and stereolithography	PCL/iron-doped HAP	Human mesenchymal stem cells	Bone tissue engineering	(90)
Electric Field	Extrusion	Graphene	Mesenchymal stem cells	Bone tissue engineering	(91)
	Inkjet	Silicone	–	Fabrication of very thin dielectric elastomer membranes used for dielectric elastomer actuator devices	(92)
	Laser sintering	Auxetic composites	–	Design of materials with programmable energy absorption capability	(93)
Biological factors	Extrusion-based	PCL, PLGA^e^, β-TCP^f^	Human nasal inferior turbinate tissue derived mesenchymal stem cells	Fabrication of tissue constructs using cell laden mineralized ECM	(94)

a PEGDA: polyethylene glycol diacrylate.

b HUVECs: human umbilical vein endothelial cells.

c Fe_3_O_4_: iron oxide.

d PCL: Poly(ε-caprolactone).

e PLGA: poly(lactic-co-glycolic acid).

f β-TCP: beta-tricalcium phosphate.

### 4.3 Applications of 4D Printing

4D printing has applications in areas such as biosensors, biorobots, bioactuators, smart biomedical devices, tissue engineering, etc. Smart biomedical devices utilize 4D printing for the manufacturing of devices using smart materials that can help track physiological changes in the patient’s body. Biosensors utilize these smart materials to track changes in the cell activity and for diagnostic purposes by tracking metabolites. Biorobots are fabricated using smart materials and help deliver therapeutic agents.^95^ 4D printing is a very promising field with great potential in tissue engineering. The key to this technology is the presence of smart materials that will help manipulate the cells and tissue in a desired and controlled manner for diagnostic and clinical purposes.

## 5 Regulatory Aspects Concerning Nanomaterials

The integration of nanomaterials in 3D bioprinting has necessitated significant changes in regulatory affairs to address the unique challenges and potential risks associated with these advanced materials. Nanomaterials, due to their small size and high surface area, exhibit distinct physical and chemical properties that can lead to oxidative stress, genotoxicity, and carcinogenicity, which are not typically observed with larger particles of the same composition.^96^ This has resulted in interest in revising existing guidelines, such as OECD 412 and OECD 413, to add some further investigations and end points within the test scheme for the assessment of risks associated with nanomaterials.^97^ On the medical device side, European Regulation 2017/745 places devices containing nanomaterials into Class III, the highest-risk category, unless the nanomaterials are encapsulated or bound to decrease internal exposure.^98^ The application of 3D bioprinting in nanotoxicology, with a focus on long-term studies involving lung cells, has proven that it is possible for 3D cultures to mimic better the *in vivo* situation than the conventionally used 2D cultures, enabling the generation of more reliable data for regulatory assessments. This progress indicates a requirement for changed regulatory frameworks, ones capable of handling the complexities brought forward by both 3D bioprinting and nanomaterials. Some critical players in developing regulation on nanotechnology include the European Union, the United States, and China, representing a fragmented yet evolving character of electronic governance globally.^99^ Further complicating this is the rapid rate at which nanomedicines under development are growing, such that guidelines for this area need to be created in a fashion that balances market success with risk assessment and safety optimization.^100^ Despite these efforts, several transnational regulatory challenges persist, including whether to adapt existing legislation or develop new frameworks, how to define nanomaterials, and how to address the limitations of current risk assessment methodologies.^101^ The European Union, for instance, has begun to incorporate nanomaterials into existing legislation for chemicals, pharmaceuticals, and medical devices, although explicit mentions of nanomaterials are still lacking.^102^ The substantial production and application of engineered nanomaterials (ENMs) have raised concerns about their environmental and human health impacts, leading to calls for compulsory reporting schemes and the development of new assessment tools such as quantitative structure–activity relationship and adverse outcome pathway (QSAR-AOP) models.^103^ The role nanomaterials play in enhancing the functionality of bioinks for 3D bioprinting strengthens the call for thorough regulatory control of these materials to ensure they meet all the physiochemical and biomechanical standards for hospital/clinical service implementation.^104^ Finally, the European Commission’s recommendation on the definition of nanomaterials and the need for harmonized assessment practices underscore the ongoing efforts to develop best practices and improve the availability of quality data for regulatory purposes.^105^ These multifaceted regulatory changes are crucial for safely harnessing the potential of nanomaterials in 3D bioprinting and other innovative applications.

## 6 Conclusion

The incorporation of nanomaterials for 3D printing holds tremendous potential and promise for revolutionizing manufacturing and production in various fields. 3D printing with nanomaterials will help in achieving the required mechanical strength like thermal and electrical conductivity and also superior biocompatibility. Because of the enhancement of such properties, highly functional and customizable products can be fabricated with better performance features. With advancement in the field, it is possible to fabricate complex geometries which also showcase precision and high resolution helping in focusing on the innovation and optimization of products. The limitations of conventional materials and manufacturing techniques can be overcome by the inclusion of nanomaterials as it offers on-demand production, rapid prototyping, and also delivering customized solutions. Innovation in synthesis, processing, and printing techniques for nanomaterials is very important for complete unlocking of full potential of nanomaterials heightened 3D printing. The synergy between both nanomaterial advancement and 3D printing technologies could pave the way for a new era of high performance and sustainable products. In this review, we also discuss 4D printing as a groundbreaking advancement in the field of additive manufacturing. It adds a temporal dimension to the traditional and conventional process of 3D printing. 4D printing holds immense potential in fields such as aerospace, healthcare, and architecture. There are several challenges that are still to be addressed including material properties and stability, the precision while fabrication, and also scalability. As the technology continues to grow, it is likely to transform the fabrication of responsive and adaptable structures that will enhance the quality of life and will be a boon to human kind.
